# The views and experiences of patients and health‐care professionals on the disclosure of adverse events: A systematic review and qualitative meta‐ethnographic synthesis

**DOI:** 10.1111/hex.13029

**Published:** 2020-02-19

**Authors:** Raabia Sattar, Judith Johnson, Rebecca Lawton

**Affiliations:** ^1^ University of Leeds and Bradford Institute for Health Research Leeds UK

**Keywords:** adverse events, disclosure, health‐care professionals, meta‐ethnography, patients, review, systematic review

## Abstract

**Objective:**

To synthesize the literature on the views and experiences of patients/family members and health‐care professionals (HCPs) on the disclosure of adverse events.

**Methods:**

Systematic review of qualitative studies. Searches were conducted in MEDLINE, Embase, PubMed, CINAHL and PsycINFO. Study quality was evaluated using the Critical Appraisal Skills Programme tool. Qualitative data were analysed using a meta‐ethnographic approach, comprising reciprocal syntheses of ‘patient’ and ‘health‐care professional’ studies, combined to form a lines‐of‐argument synthesis embodying both perspectives.

**Results:**

Fifteen studies were included in the final syntheses. The results highlighted that there is a difference in attitudes and expectations between patients and HCPs regarding the disclosure conversation. Patients/family members expressed a need for information, the importance of sincere regret and a promise of improvement. However, HCPs faced several barriers, which hindered appropriate disclosure practices. These included difficulty of disclosure in a blame culture, avoidance of litigation, lack of skills on how to conduct disclosure and inconsistent guidance. A lines‐of‐argument synthesis is presented that identified both the key elements of an ideal disclosure desired by patients and the facilitators for HCPs, which can increase the likelihood of this taking place.

**Conclusions:**

Although patients/family members and HCPs both advocate disclosure, several barriers prevent HCPs from conducting disclosure effectively. Both groups have different needs for disclosure. To meet patients’ requirements, training on disclosure for HCPs and the development of an open, transparent culture within organizations are potential areas for intervention.

## 1 INTRODUCTION

The Institute of Medicine report, To Err is Human (1999) raised awareness about the multitude of clinical errors that occur in health care.^1^ An important element in managing the consequences of clinical errors is disclosure.^2^ Adverse events are all harms to a patient occurring in the patient care setting that are not due to the underlying illness itself.^3^ Disclosure is the process by which an adverse event is communicated to the patient.^4^ Studies from developed countries have reported that adverse events occur in 0.4%‐16% of hospital admissions.^5^, ^6^, ^7^, ^8^, ^9^ Although limited research has been conducted on the occurrence of errors in developing countries, it is evident they also suffer from safety problems.^10^, ^11^

Disclosure is imperative as health‐care professionals (HCPs/HCP) have a responsibility to be open about adverse events and patients have the right to know what has happened.^12^ Disclosure maintains trust between patients and HCPs, and failure to disclose can result in increased litigation by patients^4^, ^13^, ^14^ Frameworks have been developed in several countries (UK, Australia, Canada and USA) to guide adverse event disclosure.^12^ Although transparency and openness are promoted in these policies, research suggests that disclosure does not always occur.^15^, ^16^ Research remains limited on the perspectives of patients and HCPs on adverse event disclosure. A previous comprehensive review on disclosure has been conducted^17^; however, the questions still remain about how to best disclose adverse events to patients and ways in which HCPs can be supported to meet the needs of patients. Exploring both patients’ and HCPs’ views on disclosure will help understand the expectations, barriers and challenges faced by each group. This can help to generate interventions that are effective and practical for both patients and HCPs. In this review, we aimed to synthesize the views and experiences of patients and HCPs on the disclosure of adverse events, and identify the barriers and facilitators to disclosure faced by HCPs. This is the first review to synthesize the views of patients and HCPs, using a qualitative synthesis approach. We used a meta‐ethnographic synthesis approach developed by Noblit and Hare^18^ and adopted by Britten et al^19^ and Campbell et al^20^ A meta‐ethnographic synthesis was chosen as it offers a unique systematic analysis process to provide evidence on patients’ and HCPs’ views and experiences on the disclosure of adverse events. Synthesizing qualitative studies using this approach can provide important theoretical and conceptual contributions to improve health‐care policy and practice.

## 2 METHODS

A systematic search of qualitative studies was conducted, and data from included studies were synthesized using a meta‐ethnographic approach. The review was reported according to the PRISMA guidelines.^21^ The eMERGe reporting guidance^22^ was also followed to conduct and report this meta‐ethnography.

### 2.1 Search strategy and data sources

Five electronic databases were systematically searched: MEDLINE, Embase, PubMed, CINAHL and PsycINFO (see Appendix S1). The search strategy included a combination of free‐text searching of the three main concepts being examined in this review (disclosure, incident and experience) and was developed from an existing systematic review.^21^ A comprehensive set of search strategies were used in order to identify all available studies. Searches were conducted from inception to February 2017, updated to 2018.

### 2.2 Eligibility criteria

Papers were included if they were in a health‐care setting, published in English and involved qualitative data collection and analysis. Studies focusing on breaking bad news were excluded. Grey literature was also excluded.

### 2.3 Study selection

Study selection followed PRISMA guidelines (Figure S1). Two reviewers independently screened 10% of the abstracts (RS and JJ), and Cohen's kappa statistic was used to assess inter‐rater reliability (*k* ≥ 0.7). Once inter‐rater reliability was confirmed, remaining abstracts were screened by one reviewer (RS)**.** Full texts were screened by RS, and all were double‐screened by JJ and RL. Disagreements were resolved through discussion between the three reviewers.

### 2.4 Critical appraisal

To assess study quality, the Critical Appraisal Skills Programme (CASP) qualitative research checklist was used.^23^ This tool has been previously used by published reviews of qualitative studies.^24^, ^25^, ^26^, ^27^ All studies were critically appraised, and each study was assigned a numerical score out of ten, where a higher score correlated with higher quality.^24^ The two highest ranked studies were used as index studies and were the first studies from which concepts were translated into other studies, thereby shaping the analysis.^28^ This process was carried out independently by two authors, and scores were discussed to check for consistency (RS and JJ). Disagreements were resolved through discussion. No studies were excluded because of quality of appraisal. None of the studies were rated as being ‘very low’, and a majority of the studies were rated as being of ‘high quality’. Most studies reported on the methodological framework used and provided detailed descriptions of the data analysis methods. However, authors across the studies consistently failed to report on whether the relationship between the researcher and participant was considered.

## 3 DATA EXTRACTION

Two standardized data extraction forms were developed based on a published meta‐ethnography.^26^ Descriptive data were extracted in one form by RS (study population, sample characteristics, country of origin, methods including data collection and data synthesis, and study conclusions). Key concepts or ‘second‐order constructs’ (interpretations made by the primary authors) were extracted by RS and JJ into a table in Microsoft Word, alongside the illustrative quotations from study participants (‘first‐order constructs’). To preserve the primary authors’ context and second‐order construct meaning, the authors’ own terminology and definitions were maintained. The completed forms were discussed and examined for consistency, and items were assembled into common groups prior to analysis.

## 4 DATA SYNTHESIS

A meta‐ethnographic approach was used to synthesize the findings. Meta‐ethnography provides an alternative to traditional aggregative methods of synthesis and supports the development of analytical rather than descriptive findings.^29^ Meta‐ethnography relies on a process of ‘translation’ where key concepts from one study are introduced into another, and assessed to the extent to which they can account for a particular phenomenon within a different context.^18^, ^24^ Key concepts also known as ‘second‐order’ constructs are interpretations made by authors of the included studies. During this process of translation, new interpretations are developed, which are known as ‘third‐order’ constructs. These comprise a new understanding of the phenomena under study.^24^ The synthesis involves deciding whether the studies are sufficiently similar in their focus to allow for a reciprocal translation, or if the studies refute each other, a refutational synthesis is conducted.

The synthesis process for this review consisted of three stages: (a) a reciprocal translation of the ‘patient’ studies to understand their views on the disclosure process of adverse events; (b) two reciprocal translations of the ‘HCP’ [health‐care professional] studies (i) to understand HCPs’ views on the disclosure process of adverse events and (ii) to understand the barriers to disclosure faced by HCPs; and (c) a lines‐of‐argument synthesis of all the studies to outline how patients’ and HCPs’ views differ on disclosure and how the barriers faced by HCPs may contribute towards this difference in disclosure views. This was an iterative process, and all three authors (R.S, R.L and J.J) were involved in the data synthesis.

The synthesis process began by repeatedly reading the included studies and familiarization with key concepts and metaphors. The ‘raw data’ were extracted from each study including first‐order constructs (participant quotations) and second‐order constructs (primary author interpretations). Contextual information from each study was also extracted. In order to determine how the studies were related, common concepts from studies were grouped. This was approached by gathering similar themes from studies into categories of shared meaning.^22^ The studies were sufficiently similar in their focus to allow for reciprocal translation syntheses. Reciprocal translation was approached by organizing the second‐order constructs thematically, by grouping concepts with similar meanings. The studies within each grouping were then arranged chronologically (from the highest to lowest scoring paper based on quality appraisal). The concepts within each of the groupings were compared account by account in a process similar to the method of constant comparison. During this phase, the reviewers referred back to the table of study characteristics to use as a context for comparison and the original full‐text papers. We chose to conduct a lines‐of‐argument synthesis as the concepts from the ‘patient’ and ‘HCP’ studies were not strictly contradictory in nature, but more accurately described alternative perspectives of disclosure (see Appendix S2 for further detail on methods).

## 5 RESULTS

### 5.1 Study characteristics

Fifteen studies were included (Table 1). Seven were with HCPs, 4 were with patients (including family members or the general public), and 4 included both patients (including family members or the general public) and HCPs. These were published between 2003 and 2017 and involved 1205 participants. Participants were 376 patients and family members (including 18 members of the public) and 829 HCPs. HCPs included doctors, nurses, surgeons, paediatric residents and anaesthesiologists. Studies were from Canada (two studies), USA (six studies), UK (one study), Australia (three studies), Switzerland (one study), Spain (one study) and Korea (one study).

**Table 1:** Study characteristics

Author (s)	Year	Country	Participants	Data collection method	Method of data analysis
Gallagher et al^35^	2003	USA	52 patients and 46 health‐care professionals (physicians)	Focus groups	Qualitative data analysis
Duclos et al^30^	2005	USA	16 patients	Focus groups	A combined template and organizing approach
Fein et al^43^	2005	USA	204 health‐care professionals (nurses, residents, physicians, administrators) and 36 patients	Focus groups	Qualitative data analysis
Espin et al^33^	2006	Canada	28 health‐care professionals (surgeons, nurses and anaesthesiologists) and 11 patients	Interviews	Iterative grounded theory approach
Fein et al^39^	2007	USA	204 health‐care professionals (nurses, physicians and residents)	Focus groups	Systematic approach to qualitative synthesis
Iedema et al^32^	2008	Australia	23 patients and family members	Interviews	Thematic discourse analysis
Iedema et al^34^	2008	Australia	131 health‐care staff and 23 patients	Interviews	Semantic discourse analysis
Shannon et al^15^	2009	USA	96 health‐care professionals (nurses)	Focus groups	Qualitative content analysis
Coffey et al^42^	2010	Canada	24 health‐care professionals (paediatric residents)	Focus groups	Thematic analysis
Iedema et al^31^	2011	Australia	119 patients and family members	Interviews	Discourse analysis
Mazor et al^36^	2013	USA	78 patients	Interviews	Directed content analysis
McLennan et al^37^	2016	Switzerland	18 health‐care professionals (nurses)	Interviews	Conventional content analysis
Mira et al^40^	2016	Spain	27 health‐care professionals (15 physicians and 12 nurses)	Focus groups	Qualitative data analysis
Ock et al^37^	2016	Korea	16 health‐care professionals (physicians) and 18 members of the public	Interviews and focus groups	Directed content analysis
Harrison et al^41^	2017	UK	13 doctors and 22 nurses	Interviews	Framework analysis

The following sections show reciprocal translations of ‘patient’ and ‘HCP’ studies, followed by a lines‐of‐argument synthesis (see Appendix S3 for reciprocal translation findings).

### 5.2 Reciprocal translation of patient studies

Reciprocal translation of key concepts extracted from the 8 ‘patient’ studies synthesized 3 third‐order constructs: ‘Need for information’, ‘Importance of sincere regret’ and ‘Promise of improvement’ (Table 2).

**Table 2:** Examples of reciprocal translations for ‘patients’ and ‘HCPs’

Third‐order construct	Second‐order construct	First‐order constructs
Third‐order constructs (higher order interpretations developed from a tertiary analysis of the first‐ and second‐order constructs)	Second‐order constructs (primary authors’ interpretations of the primary data—metaphorical themes or concepts)	First‐order constructs (primary data reported in each paper (participant quotations))
*Patient studies*		
Need for information	Patient frustrations^30^	‘I wanted as much…whether I understood it or not. I wanted to hear it. I wanted details because then I could sort through it in my head, and then come to my own conclusions’^30^
	Inadequate preparation for open disclosure^31^	‘We want to know what happened that day. Why was she moved from the room?..That could have contributed to her disorientation…They said oh well, we can't really give you that information’^31^
	Full disclosure^33^	‘Well it's my body, it's not the surgeon's body, and so I would want to know all the details’^33^
Importance of sincere regret	Patient frustration^30^	‘As far as just the medical people involved. That was extremely frustrating for me because nobody was willing to say that they made a mistake’ (29); ‘I just wanted him to take responsibility for it. ‘Look I’m sorry I did this and I’ll do whatever it takes to make things right’. Just own up to what happened’^30^
	Was an apology offered and of what kind?^32^	‘But it would have been nice if someone had have just acknowledged and said ‘this is our fault’…‘I definitely didn't like the defensive nature of the people involved…they were blaming the cancer’^32^
	Responsibility^36^	‘Taking responsibility, that's kind of what it's all about…it made me feel that I could trust my PCP because I mean she took responsibility…had remorse about what happened. She wasn't defensive about it…it goes a long way for me if a person can acknowledge ‘I made a mistake’ 36
	Importance of delivering an apology in open disclosure^37^	When a patient is harmed or dies, we want a whole hearted apology. Medical disputes come later on. Money and whatnot comes second… A good tongue is a good weapon, you know. With a heartfelt ‘sorry’….^37^
Promise of improvement	Need to promise recurrence prevention in ambiguous medical errors^37^	‘Well assuring recurrence prevention, this is a must, whatever the case….I’m sure when doctors say how sorry they are for what happened and reassure [the patients] that they'll make an effort to reduce possible complications, the patients will go back home feeling much better… No benefits whatsoever, but credibility will soar, I reckon’^37^
	Preventing recurrences^36^	‘The important thing is that it doesn't happen again’… ‘The point that should be made is that she knew she made a mistake and will try harder not to do that again to anybody else’^36^
	Insufficient integration of open disclosure with improvement of patient safety^31^	‘At the end of the day, you know when an unfortunate incident happens like that, that [inappropriate disclosure communication] could be avoided in the future…it would be good to know that my dad's death, you know, sort of prompted some changes in that area’^31^
*Health‐care professional studies*		
Sometimes economical with the truth	How to disclose^35^	‘I think you have to be a spin doctor all the time and put the right spin on it…I don't think you have to soft pedal the issue, but I think you have to try and put it in the best light. I think you have to be forthright with the patient to help them. And how you word it makes a big difference’^35^
	Partial disclosure^33^	‘The patient's gonna be told, but what you say about how that injury occurs depends’^33^
	Attitudes and experiences concerning disclosing errors to patients^38^	‘If I think it could have been a serious error that might have caused this damage to the patient, it will be explained differently or in a way the patient cannot realise’^38^
Owning up without saying I’m sorry	Responsibility^42^	‘I made an error. I discontinued a medication that I shouldn't have‐by accident. You know, I picked up the error, presented it to the family. You know I tried to make it a system thing because the reason I did it was not because I’m a dummy. I’m sure it could have happened to the next guy in my shoes but I felt it was my responsibility to tell the family and I did’^42^
	Support for open disclosure^34^	‘I really don't know what happened. I really can't explain what happened, but it shouldn't have happened, and I have to take the responsibility for it’^34^
	How should open disclosure be carried out^37^	‘I don't literally bring up the word regrettable but I do it eventually…it's a Korean thing that you don't really need to put it into words to…the biggest problem is when you're about to discharge your patient after stitch removal, the last step of the surgery, the wound starts to open up. It'll drive you crazy and what can you say to the patient? Seems like you can't go home today…that's the Korean way of saying sorry…you don't really need to say it through words’^37^
To tell or to not tell? When honesty may cause unnecessary anxiety Outcome determines disclosure	When should open disclosure take place^32^	‘If a patient is 95 and bed‐ridden, you might not want to tell them…it could be upsetting, they will not understand this could happen to anyone with this case.’^32^
	Attitudes and experiences concerning disclosing errors to patients^38^	‘You perceive this when dealing with patients; there are people who prefer not to know. And you need to somehow develop a sure instinct not to burden them’^38^
	Whether to disclose near misses^35^	‘My job is to relieve anxiety, not to create it. And to a certain extent when an error occurs that doesn't get to the patient, it's not their problem, it's my problem’^35^
	When should open disclosure take place^37^	‘I suppose medical errors causing minor harm will be even more problematic…Hmm I’d rather not say. This is a matter of preference I think. The patient might not feel the need either. Telling the truth is the right thing to do but since nothing really happened, I guess doctors would be inclined not to do so’^37^
	Whether to disclose near misses^35^	‘I think if we were held to disclose all of those [near misses], I think that happens so often we wouldn't have the opportunity to practise medicine’^35^
	Attitudes and experiences concerning disclosing errors to patients^38^	‘In general, the patient clearly has the right [to be informed], whether it is a small or big error. But when errors happen that have no effect on the patient, when nothing happens‐ small errors that have no effect or the patient would not see the error as an error‐ then we would not tell’^38^
*Health‐care professional studies‐ barriers to disclosure*		
Difficulty of disclosure in a blame culture	Institutional culture^43^	‘There needs to be a culture where individuals do not feel penalised for reporting errors. You should feel comfortable reporting to the chief of service of the head of nursing’^43^
	Reputation risk^42^	‘I think there's an openness about‐ we've caught that near miss. Give everybody a pat on the back whereas if something then bad happens, I think there's less of an openness and then you get more into looking at well‐rather than what the system did, you look at the people in the system’^42^
	Barriers to disclosure^38^	‘The common working culture can be beneficial or also hindering. For example, if you have to fear reprisal once you disclose an error, that this falls back on a person who is then ostracised or even loses their job’^38^
Avoidance of litigation	Understanding the repercussions^41^	‘I’ve learned that it's also quite a self‐preserving thing to do…the worst thing…is if they [patients] get it into their heads that there's some sort of cover up going on, then they get the bit between their teeth and solicitors get involved and it's all very difficult’^41^
	Reputation risk^42^	‘If families for whatever reason feel that they have not received the best medical care, they're going to make a big stink and go to the paper and feel hard done by and I think in the situations where the families are pressing and the families raising doubts‐ it may be more difficult to disclose’^42^
	Provider factors^43^	‘…two is fear of being sued and what is that going to do with your future’^43^
Disclosure is a learned skill	Absence of disclosure education^37^	I have never learned (open disclosure). Can't make facial expressions. Can't come up with words to say… ‘I have never seen anyone do it, so I have no clue on how to do it’^37^
	Role models and guidance^41^	‘I haven't had any personal training. Certainly, the trust offers a sort of day if you like around breaking bad news, however I think that tends to be more related to breaking, you know, cancers and diagnoses type thing, rather than adverse events that happened^41^
	Provider factors^43^	‘As soon as it gets into the legal realm, suddenly as an attending physician, I feel like I need to be coached as to what can be said and how it can be said and so forth’^43^
Inconsistent guidance	It all depends on your nurse manager^15^	‘She actually got a big lecture saying ‘you always run it by somebody before you disclose it to the families, because bedside nurses are not trained to discern litigiousness’…she felt like she did the right thing but was being told ‘don't do that again’^16^
	Provider factors^43^	‘The emphasis at least in my training, has been – don't talk about anything, keep quiet’^43^
	Institutional culture^43^	I can say right now that I do not know what the policy is’^43^

#### 5.2.1 Need for information

Patients felt that they were not provided with the information they needed.^30^, ^31^, ^32^, ^33^ This led to worries about what was going to happen to them next.^30^ Patients consistently emphasized the importance of receiving relevant information. However, obtaining information was problematic, difficult and time‐consuming.^34^ Patients also believed they had a right to receive information and full disclosure.^33^ Patients wanted HCPs to inform them comprehensively about the adverse event, the management plan and the investigation. Patients did not want to have to ask numerous questions of their doctor. Patients threatened legal action in order to receive information and stated it was *refreshing* when they did not have to battle with insurance companies to get information.^30^ Only in one study were patients satisfied with the information provided to them during the disclosure process and still had confidence in their HCP as a result of him being *honest*: ‘he laid it on the line and gave me the facts’.^30^

#### 5.2.2 Importance of sincere regret

A predominant theme related to the need for accountability and an apology. Patients said they expected the HCPs delivering the disclosure conversation to acknowledge what had happened and take responsibility for their actions.^30^, ^31^, ^35^, ^36^The inability to admit a mistake by HCPs and abnegating responsibility led to patient frustrations and disappointment.^30^, ^32^ When HCPs took responsibility, the patient‐professional relationship improved and there was an increased sense of trust from patients.^30^, ^32^ For some patients, assuming responsibility was seen as a pre‐requisite for learning. Patients indicated they wanted HCPs and institutions to regret what had happened:‘…it made me feel that I could trust my PCP [primary care physician] because I mean she took responsibility…had remorse about what happened’.^36^ Patients responded positively to expressions of regret and apology, but only if these were perceived to be sincere. However, for some patients, an apology alone was not sufficient; they wanted to be informed of the steps taken to correct the incident.^36^

#### 5.2.3 Promise of improvement

Patients and family members wanted to be assured that the HCPs and institution were working to prevent recurrences.^31^, ^35^, ^36^, ^37^ Patients who had suffered due to the adverse event believed it was vital that the same error was not made with other patients.^36^ An important element in preventing recurrences was that those involved had learnt from the adverse event and the incident had resulted in institutional changes.^36^ Patients specified that in disclosure conversations, they would like their HCP to state ‘we assure you this problem will not happen again’.^35^

### 5.3 Reciprocal translation of HCP studies

Reciprocal translation of the key concepts extracted from the 11 ‘HCP’ studies synthesized 3 third‐order constructs: ‘sometimes economical with the truth’, ‘owning up without saying I’m sorry’ and ‘To tell or to not tell?’ which consisted of the following two subthemes: ‘when honesty may cause unnecessary anxiety’ and ‘outcome determines disclosure’.

#### 5.3.1 Sometimes economical with the truth

A predominant theme across some of the studies related to excluding some facts or information when providing the explanation.^33^, ^35^, ^38^, ^39^, ^40^ HCPs including nurses and physicians advocated the disclosure of adverse events to patients and their families, but sometimes provided only partial and selected information to patients/ family.^33^, ^35^, ^38^, ^39^, ^40^ Many HCPs described avoiding revealing too much truth to patient or families.^33^, ^35^, ^38^, ^39^, ^40^ Errors that resulted in adverse events with a more serious outcome were disclosed in such a way that it would not be directly obvious to the patient/family that an adverse event had occurred and the fault of the HCP(s) or the institution would be concealed.^33^, ^35^, ^38^, ^39^ This was achieved in different ways. At times, the error and the adverse event were described ‘in the most positive spin and in a positive light’.^35^ Some HCPs explained the adverse event in clinical terms so that the patient/families would find it difficult to establish the connection between the error and the resulting adverse event.^38^ Others described omitting certain information related to the adverse event. 33, 38, 39

#### 5.3.2 Owning up without saying I’m sorry

Most HCPs believed an important element of disclosure was to acknowledge and accept responsibility for the adverse event.^16^, ^32^, ^34^, ^41^, ^42^, ^43^ Disclosing the event and admitting fault was considered to be a moral, ethical and professional duty.^16^, ^41^, ^43^ HCPs believed in ensuring the patient was made aware of this, even if the events leading up to the adverse event were not clear.^34^ In some situations, HCPs accepted responsibility for the adverse event; however, errors were often viewed as system faults rather than individual failures: ‘it could have been made by anyone else in my shoes’.^42^ However, in one study physicians did not explicitly disclose responsibility or express regret in specific terms.^37^ This study was conducted with HCPs from a non‐Westernized country (Korea), whereas HCPs who believed it was important to vocalize this acknowledgement to patients were based in Westernized countries. Unlike most Western countries, open disclosure policies have not yet been implemented in Korea, which may explain the difference in views.

The importance of an apology in the disclosure conversation was only cited by HCPs in one study^41^ where there was a widespread belief that an apology was not acceptance of liability and had no professional and legal implications. However, this group described the existence previously of a culture where apologizing was considered to be an admission of liability.^41^ This culture may still exist elsewhere, and this could explain why apologizing was not discussed by HCPs in other studies. Conversely, some doctors believed that it was not necessary to state an apology as ‘you don't really need to say it through words’.^37^

#### 5.3.3 To tell or to not tell?

Within this theme, the data were fractured into two subthemes: ‘when honesty may cause unnecessary anxiety’ and ‘outcome determines disclosure’.

#### 5.3.4 When honesty may cause unnecessary anxiety

Health‐care professionals assumed in cases where the error was not obvious or evident, the patient would rather not be informed.^33^, ^35^, ^37^, ^38^ Where it was felt the patient would be burdened by the disclosure, or it would cause unnecessary stress, the event was not disclosed: ‘if a patient is 95 and bed‐ridden‐ you might not want to tell them’.^33^ One HCP related ‘My job is to relieve anxiety, not to create it’.^35^ Non‐disclosure in such cases was rationalized as being in the patients best interests.

#### 5.3.5 Outcome determines disclosure

The impact of an error on the patient, whether it resulted in an adverse event, and the severity of the adverse event influenced whether disclosure occurred. When adverse events were minor or there was no substantial harm resulting from errors, disclosure was not believed to be of importance^33^, ^35^, ^37^, ^38^, ^39^, ^43^ and was seen as impractical due to the frequent occurrence of these events.^35^ Lack of patient awareness of the error was also considered as a reason for non‐disclosure.^35^, ^38^, ^43^ Disclosure of medical errors resulting in minor or no adverse events was compared to aviation errors by suggesting that near misses occur frequently in aviation; however, people would only be informed if they became aware of these.^43^ Conversely, one HCP endorsed the disclosure of errors, which did not lead to adverse events, and saw this as an opportunity to improve their trust with patients.^35^

### 5.4 Reciprocal translation of HCP studies on barriers to disclosure

Reciprocal translation of the key concepts on the barriers to disclosure synthesized 4 third‐order constructs: ‘difficulty of disclosure in a blame culture’, ‘avoidance of litigation’, ‘disclosure is a learned skill’ and ‘inconsistent guidance’. These barriers described by HCPs may help to explain the differences between the two group's perspectives on disclosure.

#### 5.4.1 Difficulty of disclosure in a blame culture

One of the commonly cited barriers to disclosing adverse events was a culture of blame, where these cultural barriers were either organizational 35, 38, 41, 43 or professional in origin.^38^, ^41^, ^42^, ^43^ At times, it was difficult to distinguish between what constitutes as an organizational and professional/workplace barrier. Nurses, in particular, described their health‐care organizations as having a closed culture, which inhibited openness with patients in relation to adverse events.^38^, ^43^ Non‐disclosure was attributed to the institutional culture in which the HCPs were immersed.^43^ When adverse events occurred, blame was attributed towards individuals’ errors rather than system errors, which resulted in HCPs being apprehensive to disclose.^42^ Changes in the institutional culture were suggested by nurses, doctors and HCPs as a strategy to promote disclosure practices. This included the removal of a blame culture and the development of a culture of openness and transparency.^43^

Support and guidance from managers was described by nurses as a factor, which either positively or negatively influenced disclosure practices.^16^, ^43^ Supportive managers were described as never attributing blame to the individual but being empathetic and understanding as ‘this could happen to anybody’.^16^ On the other hand, HCPs conveyed that they would be less likely to disclose future adverse events if they believed they had been unfairly blamed or shamed in the past. One nurse provided an account where a colleague was reprimanded for disclosing an adverse event to a patient, leading to the belief that they were being discouraged from carrying out their professional and moral duty to inform the patient.^16^ In this situation, ‘she felt like she did the right thing, but was told, ‘don't do that again’.^16^

Worries and fear that a damaged reputation will accompany admittances of adverse events also hindered disclosure practices.^35^, ^38^, ^42^ HCPs described fear of admitting and openly disclosing adverse events due to worries about how they would be perceived by colleagues and whether their mistakes would be discussed within the organization.^43^ This was more so for the junior clinicians as they believed that there was an expectation to prove their competence.^42^ Concerns about whether their mistakes would be discussed within the organization were also voiced.^42^

Fear of professional or workplace sanctions was also described as one of the reasons for concealing adverse events.^38^, ^41^, ^42^, ^43^ This included being fearful of disciplinary action that may be taken against them including losing their job,^38^, ^41^ fear of being ostracized within their workplace^38^ and fear of damaging career opportunities expressed by physicians in particular.^35^, ^42^ These sanctions that were put in place were found to be counterproductive in responding and disclosing future adverse events.^38^

#### 5.4.2 Avoidance of litigation

A second category of barriers to disclosure comprised of fears about exposure to legal liability because of admitting and disclosing an adverse event to patients.^30^, ^33^, ^38^, ^41^, ^42^ HCPs described worries that patient/family would take legal action against them if they took responsibility and were open and transparent about the adverse event.^35^, ^41^, ^42^, ^43^ HCP cited concerns that disclosure would result in patients/or family causing further issues if they felt that they had not received the best medical care.^42^ Furthermore, HCPs were apprehensive about including an apology in the disclosure conversation as there was the belief that it may be considered as an admission of legal liability.^35^ The HCPs expressed a desire to be ‘just straightforward’ 35 about the adverse incident and the events that led to it, however, believed that in reality, this would result in a lawsuit.

HCPs from one UK study had contrasting views to this where they held the belief that patients/families would have a more ‘generous and understanding view’ of the adverse event if they were honest and upfront about the situation.^41^ These HCPs advocated disclosure and transparency and described witnessing adverse events, which resulted in harm to the patient, but legal action was not pursued due to open disclosure.^41^ The differences in HCPs’ views in this study may have been influenced by the recent duty of candour regulation, which was implemented in the UK two years prior to this study. Some HCPs acknowledged litigation fears as a barrier to disclosure, but regardless of these fears, they believed disclosure enhanced the patient‐provider relationship and was therefore considered as valuable.^41^

#### 5.4.3 Disclosure is a learned skill

HCPs described that a lack of training or the absence of disclosure education led to a lack of confidence in skills, which resulted in hindered disclosure practices.^37^, ^38^, ^41^, ^43^ The value of open disclosure education was recognized; however, one HCP related ‘I haven't had any personal training’.^41^ Similarly, other HCPs reinforced the importance of training specific to disclosing adverse events, but stated that they had only received training on how to break bad news to patients and had not received any training specific to adverse event disclosure.^37^, ^38^, ^43^ HCPs believed that they lacked skills on how to communicate certain aspects of the adverse events. This lack of skills led to a lack of confidence when conducting the disclosure.^43^ No training was provided from a legal perspective, and HCPs believed when it came to the legal aspects, they required more educational support.^43^ A situation was described where the HCP was remorseful about what had happened; however, he ‘just didn't know how to express or convey it’.^37^ Regardless of which country HCPs belonged to, a distinct need for education and training in this area was expressed by HCPs,^37^, ^38^, ^41^, ^43^ suggesting that a lack of training is a universal issue.

#### 5.4.4 Inconsistent guidance

HCPs described that there was a lack of clarity on what should be disclosed, when and how, as they were provided with varying guidance from seniors or management within their organization.^16^, ^38^, ^41^, ^43^ HCPs were provided with contradictory views on whether they should disclose the adverse event. Instances were described where HCPs were asked to refrain from disclosing the adverse event to colleagues or the patient/family.^16^, ^41^, ^43^ Lack of awareness of organizational policies related to adverse event disclosure also made this process challenging.^16^, ^43^ Some HCPs were simply unaware of the existence of these policies, whereas others suggested the policy needed to be made known and enforced in the organization as the existence of a policy alone did not ensure that appropriate disclosure practices would take place.^42^ Furthermore, some HCPs held the belief that awareness and implementation of organizational policies could promote openness if guidelines for disclosure were provided.^16^

### 5.5 Lines‐of‐argument synthesis

The syntheses of ‘patient’ and ‘health‐care professional’ (HCP/HCPs) studies in this review revealed that there was a disconnect between the perspectives of these two groups on how disclosure should be conducted and what the disclosure conversation should entail. These key differences are discussed in relation to the barriers to disclosure faced by HCPs.

There was a difference between patients and HCPs in their expectations and attitudes about what the disclosure conversation should include. HCPs of different clinical professions had similar perspectives regarding disclosure. Although only two studies included family members,^31^, ^32^ their views were in agreement with those of patients. Patients emphasized their right to receive all the relevant information related to the adverse event, but described not receiving the information they needed from HCPs.^30^, ^31^, ^32^, ^33^ HCPs also acknowledged the importance of disclosure but said that often they would omit some information from the conversation.^33^, ^35^, ^38^, ^39^, ^40^ A majority of the time, HCPs did not provide patients with the breadth of information they required. This could be explained by the barriers HCPs were faced with. They described worries that they would be exposed to litigation if they were open and transparent with patients.^35^, ^38^, ^41^, ^42^, ^43^ Some expressed their desire to be *straightforward* but believed this would result in a lawsuit.^35^ There has recently been more awareness that open disclosure does not necessarily increase litigation risk, which was reflected by HCPs in one of the later studies conducted in the UK.^41^ Within this study, HCPs acknowledged that the issue of litigation did exist, but believed that patients had a more *generous* and *understanding view* if they were honest and transparent. These professionals also described witnessing adverse events, which resulted in harm, but which did not result in patients pursuing legal action due to the practice of open disclosure.

Apologizing and taking responsibility was an element that many patients/family members considered to be an important aspect of disclosure.^30^, ^31^, ^32^ Many patients wanted an apology from the HCPs as they felt this signified an expression of regret. However, HCPs from only one study cited the importance of including an apology in the disclosure conversation. Some professionals were worried that an apology would be interpreted as evidence, which could be used to prove legal liability. There has been a previous existence of a culture where apologizing was considered as an admission of legal liability, and it is possible this culture still existed whilst most of these studies were conducted (between 2003 and 2017). Patients also wanted assurance that recurrences of the same adverse event would be prevented^31^, ^35^, ^36^, ^37^; however, the importance of this element was not acknowledged by HCPs in the included studies.

Finally, a lack of certainty, skill and confidence about how to disclose adverse events may have prevented many HCPs from delivering a disclosure, which included all the elements patients expressed a desire for. Regardless of clinical profession, many HCPs had not received training on how to effectively communicate the adverse event to patients,^37^, ^38^, ^41^, ^43^ which could have contributed to the gap between the information patients felt they needed and the information HCPs delivered in practice.

**Figure 1:**
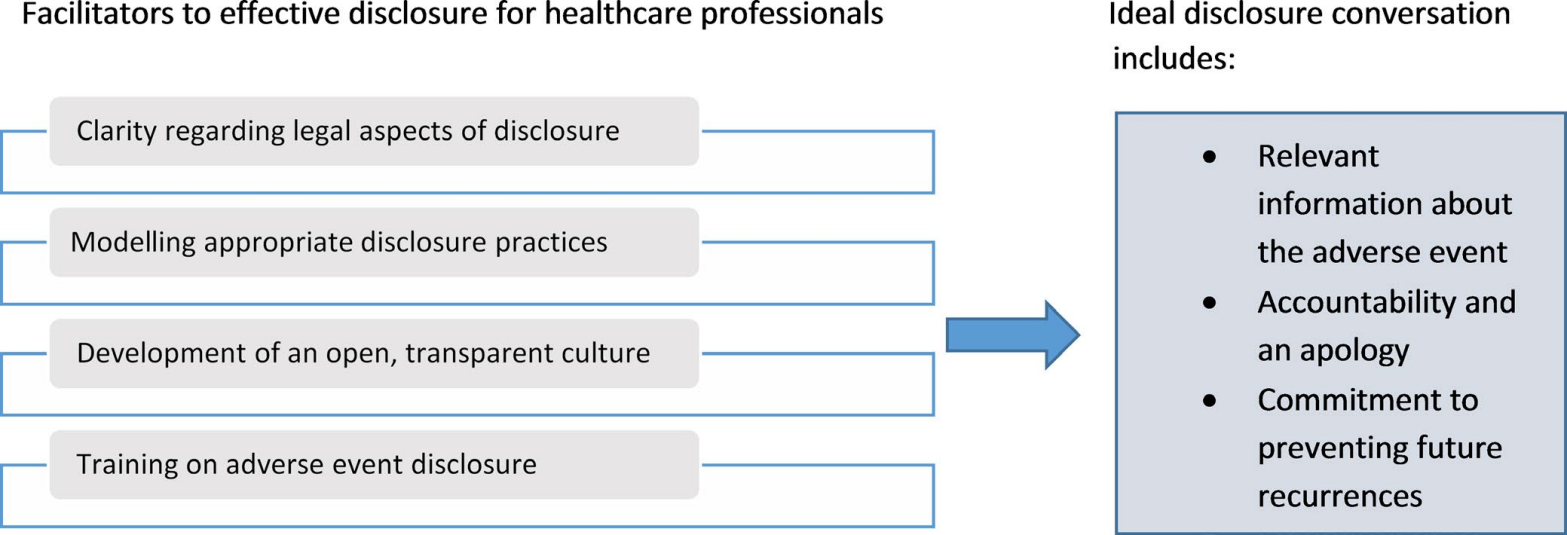
Ideal disclosure practice and facilitators to effective and practicable disclosure for health‐care professionals

Altogether, this lines‐of‐argument synthesis identified both the key elements of an ideal disclosure conversation desired by patients and the facilitators for HCPs, which can increase the likelihood of this taking place (Figure 1).

## 6 DISCUSSION

### 6.1 Summary of results

This meta‐ethnographic synthesis highlighted that there is a difference in attitudes and expectations between patients and HCPs regarding the disclosure conversation. Patients and families advocated disclosure following an adverse event. They expressed a need for certain information including accountability and an apology, and a commitment to prevent the same adverse event from occurring again. However, a majority of the time, patients did not feel satisfied with the disclosure or felt they were provided with partial disclosure, which did not include all the elements they desired.^30^, ^31^, ^33^, ^38^, ^40^ HCPs considered disclosure to be a moral and professional duty^16^, ^41^, ^43^; however, multiple barriers prevented them from carrying out this disclosure. These include an organizational culture of blame, litigation fears and lack of skills and training on how to conduct disclosure. The findings of this review suggest that there is an evident gap between the expected communication practice and what is being done.

### 6.2 Strengths and limitations

Strengths of meta‐ethnographic approaches include their preservation of the interpretative properties of the primary data^44^ and their potential to provide higher levels of analysis.^28^ Meta‐ethnography is inevitably limited by the breadth and quality of studies^24^; the articles included within this review were identified using a systematic approach, which enabled the identification of all relevant studies published within the area of disclosure. Although a comprehensive systematic search was undertaken, it is possible that not all the relevant studies were retrieved. The necessary inclusion of studies from different countries could be considered to be a further limitation as each country has a different health‐care system, thereby making the transferability of findings difficult. Also, due to the different health‐care systems represented in the literature, there is a lack of transferability of the legal aspects of disclosure. Therefore, the legal barriers to disclosure perceived by HCPs may vary accordingly across countries. Also, a potential limitation is that all of the included patient studies were conducted in Westernized countries including the USA, Australia and Canada. Therefore, it can be argued that the literature represents the views and cultural expectations of a Westernized culture. One of the reasons why a majority of the research may be conducted in Western countries is that there are laws and policies in place in these countries, which require patients to be informed of all aspects of their care, including any unanticipated outcomes.

### 6.3 Implications for clinicians, policymakers and researchers

This review has highlighted that there is a gap between what patients desire from disclosure and what HCPs offer. The lines‐of‐argument synthesis highlighted the following ways in which this gap can be reduced. At an organizational level, there is a need to develop consistent and transparent policies and promote and enforce these within organizations.^16^, ^38^, ^41^, ^43^ Also, shifting away from the existence of a blame culture and the development of an open and transparent culture can help facilitate effective disclosure practices.^35^, ^38^, ^41^, ^42^, ^43^ One of the frequently cited barriers to disclosure was fear of litigation. However, patients described that they sought legal help when they did not receive appropriate disclosure (eg HCPs failed to apologize or take responsibility for the adverse event). Patients did not desire to punish HCPs or collect large sums of money. Therefore, it is imperative that HCPs receive education regarding the legal aspects of disclosure, including the legal protections that are currently in place and reasons why patients seek legal support.

NICE (National Institute for Health and Care Excellence), which provides national guidance and advice to improve health and social care within the UK, highlights the importance of HCPs being proficient in communication skills,^45^ and disclosure is a complex communication task. The findings of this review showed that HCPs required more adverse event disclosure training.^37^, ^38^, ^41^, ^43^ A number of studies have developed and discussed adverse event disclosure educational programmes, which have used a variety of teaching methods.^46^, ^47^, ^48^, ^49^, ^50^, ^51^ However at an organizational level, these disclosure educational programmes need to be integrated into training programmes for HCPs.

HCPs also expressed worry and anxiety about disclosure. These worries stemmed from a blame culture, which surrounded health‐care organizations, risk of damaging reputation and fear of litigation.^35^, ^38^, ^41^, ^42^, ^43^ More clarity and guidance is needed on what constitutes relevant information that is provided during disclosure, as patients and HCPs have different perceptions regarding this. HCPs also held inaccurate assumptions about what patients/family members would want to be disclosed.^33^, ^35^, ^37^, ^38^ Providing HCPs with disclosure training that is specific to adverse events is one of the potential ways to improve this process. Training interventions should be informed by the elements patients desire from disclosure and guidance from recent disclosure policies. Educating HCPs on the benefits of taking a patient‐centred approach when disclosing adverse events is one of the potential ways to meet patients’ needs. This would include thinking beyond the HCPs’ own beliefs and deem what is important for patients. Involving patients/family members in the adverse event investigation is another one of the potential ways to meet the needs of this group.

## 7 CONCLUSION

This is the first qualitative meta‐ethnography of patients’ and HCPs’ experiences of adverse event disclosure. Our findings suggest that although patients and HCPs both advocate disclosure, a number of barriers prevent HCPs from carrying out disclosure effectively. HCPs also hold inaccurate beliefs about when and what patients want to be disclosed. To meet patients’ needs for disclosure, training on disclosure for HCPs and the development of an open, transparent culture within organizations are potential areas for intervention. Also, the responses of patients and relatives are not fully predictable and even the best of open disclosure practices may not resolve their problems or concerns. Therefore, it is important that guidance and training for HCPs reflect these challenges.

## CONFLICT OF INTEREST

None declared.

## Data Availability

The data that support the findings of this study are available from the corresponding author upon reasonable request.
