# Supplementary material for: The views and experiences of patients and health‐care professionals on the disclosure of adverse events: A systematic review and qualitative meta‐ethnographic synthesis
Source: Health Expect. 2020 Feb 19;23(3):571–83. doi: 10.1111/hex.13029 (PMC7321730; doi:10.1111/hex.13029)
Supplement: Supplementary file 1 [file HEX-23-571-s001.docx]

Online supplementary appendix 1: Searches from 5 electronic databases

**Ovid Medline** *(1996 -January week 4 2018)*

1. Disclosure* 29718
2. Truth disclosure* 9248
3. Open disclosure* 68
4. Doctor patient-relation* 2019

5. #1 OR #2 OR #3 OR #4 31626

6. Medical error* 15641
7. Medication error* 9881
8. Diagnostic error* 19124
9. Iatrogenic disease* 7695
10. Adverse event* 93325
11. Patient safety* 24335
12.Patient safety incident* 205
13. Serious untoward event* 2
14. Risk management* 17239
15. Healthcare error* 21
16. Surgical error* 243
17. Therapeutic error* 131

18. #6 OR #7 OR #8 OR #9 OR #10 OR #11 OR #12 OR #13 OR #14 OR #15 OR #16 OR #17 = 173212

19. Attitude* 251828
20. Perception* 232179
21. Preference* 89717
22. Views* 39495
23. Experience*
24. Perspective 550283

24. #19 OR #20 OR #21 OR #22 OR #23= 1017250

#5 AND #18 AND #24= 453

**Embase (**1996 to 2018 week 06)

1. Disclosure* 21396
2. Truth disclosure* 116
3. Open disclosure* 86
4. Doctor patient-relation* 91627

5. #1OR #2 OR #3 OR #4 = 111636

6. Medical error* 18107
7. Medication error* 15336
8. Diagnostic error* 42556
9. Iatrogenic disease* 19139
10. Adverse event* 186139
11. Patient safety* 93723
12. Patient safety incident* 341
13. Serious untoward event* 7
14. Risk management* 37966
15. Healthcare error* 24
16. Surgical error* 1438
17. Therapeutic error* 1429

18. #6 OR #7 OR #8 OR #9 OR #10 OR #11 OR #12 OR #13 OR #14 OR #15 OR #16 OR #17 = 389002

19. Attitude* 343537
20. Perception* 275475
21. Preference* 132408
22. Views* 60618
23. Experience* 940569

24. #19 OR #20 OR #21 OR #22 OR #23= 1561664

#5 AND #18 AND #24= 1966

**PubMed** *(Inception- 03/02/18)*

1. Disclosure* 40628
2. Truth disclosure* 12472
3. Open disclosure* 80
4. Doctor patient-relation* 3322

5. #1OR #2 OR #3 OR #4 = 43779

6. Medical error* 16187
7. Medication error* 13045
8. Diagnostic error* 35415
9. Iatrogenic disease* 14933
10. Adverse event* 109564
11. Patient safety* 30287
12. Patient safety incident* 271
13. Serious untoward event* 4
14. Risk management* 23667
15. Healthcare error* 22
16. Surgical error* 395
17. Therapeutic error* 257

18. #6 OR #7 OR #8 OR #9 OR #10 OR #11 OR #12 OR #13 OR #14 OR #15 OR #16 OR #17 = 227341

19. Attitude* 354778
20. Perception* 354346
21. Preference* 128512
22. Views* 61947
23. Experience* 848772

24. #19 OR #20 OR #21 OR #22 OR #23= 1561690

#5 AND #18 AND #24= 520

**Psychinfo (***2002 to January week 5 2018)*

1. Disclosure* 11003
2. Truth disclosure* 9
3. Open disclosure* 34
4. Doctor patient-relation* 1070

5. #1OR #2 OR #3 OR #4 =12043

6. Medical error* 625
7. Medication error* 438
8. Diagnostic error* 220
9. Iatrogenic disease* 15
10. Adverse event* 8737
11. Patient safety* 2677
12. Patient safety incident* 30
13. Serious untoward event* 3
14. Risk management* 5393
15. Healthcare error* 9
16. Surgical error* 17
17. Therapeutic error* 23

18. #6 OR #7 OR #8 OR #9 OR #10 OR #11 OR #12 OR #13 OR #14 OR #15 OR #16 OR #17 = 17276

19. Attitude* 201792
20. Perception* 216972
21. Preference* 55345
22. Views* 36400
23. Experience* 365710

24. #19 OR #20 OR #21 OR #22 OR #23= 705603

#5 AND #18 AND #24= 109

**CINAHL**

1. Disclosure* 12,181
2. Truth disclosure* 7,295
3. Open disclosure* 68
4. Doctor patient-relation* 622

5. #1OR #2 OR #3 OR #4 = 12,771

6. Medical error* 1, 989
7. Medication error* 9, 594
8. Diagnostic error* 5, 696
9. Iatrogenic disease* 1, 581
10. Adverse event* 28, 610
11. Patient safety* 41, 498
12. Patient safety incident* 185
13. Serious untoward event* 1
14. Risk management* 14, 054
15. Healthcare error* 217
16. Surgical error* 152
17. Therapeutic error* 49

18. #6 OR #7 OR #8 OR #9 OR #10 OR #11 OR #12 OR #13 OR #14 OR #15 OR #16 OR #17 = 92, 142

19. Attitude* 174, 030
20. Perception* 71, 479
21. Preference* 17, 149
22. Views* 17, 689
23. Experience* 190, 500

24. #19 OR #20 OR #21 OR #22 OR #23= 384, 712

#5 AND #18 AND #24= 222
