# Supplementary material for: The views and experiences of patients and health‐care professionals on the disclosure of adverse events: A systematic review and qualitative meta‐ethnographic synthesis
Source: Health Expect. 2020 Feb 19;23(3):571–83. doi: 10.1111/hex.13029 (PMC7321730; doi:10.1111/hex.13029)
Supplement: Supplementary file 2 [file HEX-23-571-s002.docx]

Supplementary appendix 2: Data synthesis methods - A meta-ethnographic approach

This approach to analysis begins with noting verbatim and coded text in terms of first order and second order constructs, translation of constructs across the papers included in the synthesis to form third order constructs, and conducting a synthesis using either reciprocal, refutational or line of argument synthesis. When referring to first, second and third order constructs the definitions used by Britten et al [18] were utilised. First order constructs represent the primary data reported in each paper (participant quotations), second order constructs are the authors interpretations of the primary data (often metaphorical themes or concepts), and third order constructs are defined as the reviewers higher order interpretations developed from a tertiary analysis of the first and second order constructs. The synthesis process for this review consisted of three stages: (i) a reciprocal translation of the ‘patient’ studies to understand their views on the disclosure process of adverse events; (ii) a reciprocal translation of the ‘healthcare professional’ studies to understand their views on the disclosure process of adverse events; and (iii) a line of argument synthesis of all the studies to outline how patients and healthcare professionals views differ on disclosure and how the barriers faced healthcare professionals may contribute towards this difference in disclosure views. A line of argument translation was chosen as it became apparent during the synthesis that the concepts from the ‘patient’ studies and ‘healthcare professional’ studies were not strictly contradictory in nature, rather described alternative perspectives of the same phenomenon.
