# Supplementary material for: The views and experiences of patients and health‐care professionals on the disclosure of adverse events: A systematic review and qualitative meta‐ethnographic synthesis
Source: Health Expect. 2020 Feb 19;23(3):571–83. doi: 10.1111/hex.13029 (PMC7321730; doi:10.1111/hex.13029)
Supplement: Supplementary file 3 [file HEX-23-571-s003.doc]

Supplementary appendix 3: Reciprocal translation findings

Table 1: Reciprocal translation table of findings on patients views of disclosure

| Descriptor  (Groups of similar concepts clustered together/ broad thematic headings) | First order data | Second order themes |
| --- | --- | --- |
| Information provided during disclosure | ‘Well assuring recurrence prevention, this is a must, whatever the case….I’m sure when doctors say how sorry they are for what happened and reassure [the patients] that they’ll make an effort to reduce possible complications, the patients will go back home feeling much better... No benefits whatsoever, but credibility will soar, I reckon’ ^37^; ‘I still see him even though my insurance has changed and he’s no longer on my insurance. I have total confidence in him…well, he’s honest. You know he laid it on the line and gave me the facts’ ^30^; ‘I’ve been really impressed with the time he took to explain in a straightforward manner’ ^36^; ‘The important thing is that it doesn’t happen again’... ‘The point that should be made is that she knew she made a mistake and will try harder not to do that again to anybody else’… ‘Well I think she should have gone further in trying to figure out what did happen, because he does surgery all the time. He could endanger someone else’s life’ ^36^; ‘…That was extremely frustrating for me because nobody was willing to say that’s they made a mistake’ ^30^; …’Not knowing what’s really going to happen, not knowing if I’m ever going to come home again’…I was on antibiotics for weeks and they discontinued it because the antibiotics were affecting my kidneys. And it’s like well, what else is going to happen to me now? It just seemed like one thing after another after another after another’^30^ ; ‘I wanted as much…whether I understood it or not. I wanted to hear it. I wanted details because then I could sort through it in my head, and then come to my own conclusions’… ‘It’s refreshing to not be battling with insurance companies, hospitals, etc. we didn’t have to get a lawyer’ ^30^; ‘We want to know what happened that day. Why was she moved from the room?...That could have contributed to her disorientation…They said oh well, we can’t really give you that information’ ^31^; ‘After a week, ten days, the three of us decided that we wanted to see the notes, we…wanted to know what was going to happen to him…I rang up the GP…It took him ten days and then he called me’ ^31^; ‘I’ve talked and talked and talked. I’ve tried to talk to counsellors and I’ve talked to midwives, I’ve talked to doctors and I’ve talked to a lot of people…and then finally yeah eighteen months, no fifteen months later we finally had the open disclosure’ ^32^; ‘At the end of the day, you know when an unfortunate incident happens like that, that [inappropriate disclosure communication] could be avoided in the future…it would be good to know that my dad’s death, you know, sort of prompted some changes in that area’ ^31^ ; ‘I didn’t receive the opportunity to follow up and try to understand the whole’ ^32^ ; ‘…when we did go to the meeting [the patient liason officer] said he’d like to shut up and let me talk…he asked me… ‘What do you want to get out of it?’ And basically my answers were I wanted to make sure that it never happened again…and it was really good because [the liaison officer] allowed me to say that…I liked that I could talk and ask questions’ ^34^; ‘Well it’s my body, it’s not the surgeon’s body, and so I would want to know all the details’ ^33^; ‘I’m sorry but due to an error of writing instructions and communication there was a misunderstanding and it caused an overdose of insulin. You have my deepest sympathy as far as the physical problems we caused for you. However, we’re doing everything within our powers to correct this error and we can assure you this problem will not happen again because I’m not only going to address it as far as writing information down, but I’m also going to communicate it so the nurse will understand what is supposed to be given…I’m available to sit down and discuss with you in detail what happened and again, I’m sorry (patient describes how he would like the physician to describe an error)^35^ | Need to promise recurrence prevention in ambiguous medical errors^37^; Communication^30^; Provide information on what happened ^36^ ; Preventing recurrences^36^; Patient frustrations^30^; Patient worries^30^; Inadequate preparation for open disclosure^31^; Insufficient integration of open disclosure with improvement of patient safety^31^;  Was the incident promptly disclosed to the patient and/ or family? ^32^; How formal was the disclosure meeting and did it match the patient’s expectations? ^32^; Suggestions of ways to optimise the experience^34^; Trust^33^; What information to disclose about the error^35^. |
| Taking responsibility | ‘…As far as just the medical people involved. That was extremely frustrating for me because nobody was willing to say that they made a mistake’^29^; ‘I just wanted him to take responsibility for it. ‘Look I’m sorry I did this and I’ll do whatever it takes to make things right’. Just own up to what happened’ ^30^;  ‘…But it would have been nice if someone had have just acknowledged and said ‘this is our fault’ ^32^; ‘I definitely didn’t like the defensive nature of the people involved’ ^32^ ; ‘They were blaming the cancer’ ^32^; ‘…taking responsibility, that’s kind of what it’s all about’ ^36^; ‘…it made me feel that I could trust my PCP because I mean she took responsibility…had remorse about what happened. She wasn’t defensive about it’ ^36^; ‘…it goes a long way for me if a person can acknowledge ‘I made a mistake’ ^36^.  ‘When a patient is harmed or dies, we want a whole hearted apology. Medical disputes come later on. Money and whatnot comes second’ ^37^; ‘A good tongue is a good weapon, you know. With a heartfelt ‘sorry’…’ ^37^; ‘But if doctors take responsibility and apologise for my loss, I would take it on my own shoulders even where I have to earn a living for myself. Because I accepted the apology’ ^37^; ‘When such a case arises, it’s saying sorry for what’s happened and any wrongdoing possibly caused by medical staff for example. And it doesn’t necessarily mean you are admitting negligence’ ^37^; ‘…the first meeting she apologised…then when I handed her the birth certificate that said staphylococcal chorioamionitis she backtracked and said ‘no, no, no…’ and that to me seemed like she just completely and utterly not sorry anymore’ ^32^ ; ‘The fact that that had happened to me’ ^36^; ‘I think she did the right thing…she acknowledged that I’d been through a pretty terrible experience’ ^36^; ‘…trying to explain it to me, I don’t know if that would have helped any. I think what I really wanted to was someone to care, to say ‘Oh I am so sorry…’ ^36^; ‘There’s got to be accountability. I don’t want to hear ‘I’m sorry’. ‘I’m sorry’ is nothing. I want to know what steps have you taken to correct the problem? Don’t tell me you were sorry that the problem occurred. That just puts a band aid on something…I want to see results’.^36^ | Patient frustrations ^30^; Was an apology offered and of what kind? ^32^; Responsibility ^32^.  Importance of delivering an apology in open disclosure ^37^; Apology not regarded as admitting negligence ^37^; What kind of apology was offered? ^32^ Apology & expressions of regret ^36^; Importance of action ^36^ |

Table 2: Reciprocal translation table of findings on healthcare professionals views of disclosure

| Descriptor  (Groups of similar concepts clustered together/ broad thematic headings) | First order data | Second order themes |
| --- | --- | --- |
| Information provided during disclosure | ‘Whatever the case, you should give an explanation to the caregivers. On why you did it and how things can go wrong... ‘Well isn’t it natural for them to want to know? All of a sudden, a patient dies after a surgery. Sure, the odds of death from a surgical complications, one in a million and whatnot, are well known. But they probably wouldn’t have imagined anything like it happening in their own life not in a million years…’it’s designated in medical ethics, and aren’t we mandated to inform the caregivers because it’s just right?’ ^37^; ‘That is why we always go back and inform the patient. And we also always tell them exactly what we do next, so that the error does not happen again. The same applies also for the relatives. So far this has always gone well’ ^38^; ‘If I think it could have been a serious error that might have caused this damage to the patient, it will be explained differently or in a way the patient cannot realise’ ^38^; ‘I think the way it should happen in real life is that the doctor would go in and start with what happened ‘You had a seizure, you fell out of bed, you broke your hip.’ ‘Why is that?’ ‘Well it seems like that your insulin dose lowered your blood sugar and you weren’t getting any food’ and answer any questions that occur. That I think, would be full disclosure without going in and wringing your hands’ ^39^;  ‘I think you have to be a spin doctor all the time and put the right spin on it…I don’t think you have to soft pedal the issue, but I think you have to try and put it in the best light. I think you have to be forthright with the patient to help them. And how you word it makes a big difference’ ^35^; ‘Everything you read and everything that you’re told says that you are supposed to tell what errors you make as soon as you can. Let them know what you’re thinking is, what you are going to do about it. And your chances of having an adverse litigation are less if you take that approach. Now the question is, how many of us believe that?’ ^35^. ‘The patient’s gonna be told, but what you say about how that injury occurs depends’ ^33^; ‘The patient should always be informed’; ‘It is the patients right’; ‘It is more likely for them to end up in a claim for damages or litigation against the health care professionals’ ^40^. | What should be delivered through open disclosure ^37^; Attitudes and experiences concerning disclosing errors to patients ^38^; Connect the dots ^39^; How to disclose ^35^; Trust ^33^; What information to give^40^ |
| Acknowledging responsibility and apology | ‘If I’d made a mistake I’ve got to go and see that person and say look I am sorry, it was my fault. I am not saying it was right, you know it was me that did it and I did it and it was an error and I apologise. And if they then want to take that further, well that is their prerogative’ ^41^; ‘I made an error. I discontinued a medication that I shouldn’t have-by accident. You know, I picked up the error, presented it to the family. You know I tried to make it a system thing because the reason I did it was not because I’m a dummy. I’m sure it could have happened to the next guy in my shoes ^42^; ‘But I felt it was my responsibility to tell the family and I did’ ^42^; ‘If I made a mistake, then it would be my responsibility to tell them [patient or family] ^16^; ‘No matter what happens in the care of the patient…I am the one who is responsible for that patient’ ^43^; ‘It is your obligation to do so’ ^43^; ‘ I really don’t know what happened. I really can’t explain what happened, but it shouldn’t have happened, and I have to take the responsibility for it’ ^34^; ‘I don’t literally bring up the word regrettable but I do it eventually…it’s a Korean thing that you don’t really need to put it into words to…the biggest problem is when you’re about to discharge your patient after stitch removal, the last step of the surgery, the wound starts to open up. It’ll drive you crazy and what can you say to the patient? Seems like you can’t go home today…that’s the Korean way of saying sorry…you don’t really need to say it through words’ ^37^; …And if somebody has made an error or I’ve made an error then I’m going to apologise. ^41^ | Open disclosure as a moral and professional duty ^41^; Responsibility ^42^; Social context in which the participant observed or experienced the error ^42^; Who should tell the patient? ^16^; Influences on the decision to disclose a medical error^43^; Support for open disclosure^34^; How should open disclosure be carried out ^37^; Clarity ^41^ |
| When should an error be disclosed? | ‘I suppose medical errors causing minor harm will be even more problematic…Hmm I’d rather not say. This is a matter of preference I think. The patient might not feel the need either. Telling the truth is the right thing to do but since nothing really happened, I guess doctors would be inclined not to do so’ ^37^; ‘If I were a patient, I’d rather not know’ ^37^; ‘In general, the patient clearly has the right [to be informed], whether it is a small or big error. But when errors happen that have no effect on the patient, when nothing happens- small errors that have no effect or the patient would not see the error as an error- then we would not tell’ ^38^; ‘You perceive this when dealing with patients; there are people who prefer not to know. And you need to somehow develop a sure instinct not to burden them’ ^38^; ‘...It’s probably once a month or more often we’ll have some old person come in with a massive intracranial fatal haemorrhage, and the INR is 4 or 6 or 10. We don’t tell them’ ^39^; ‘there was once case that we had, a guy who got two doses of Lovenox back to back. Nursing error. And then he had a… haemorrhage and went to the unit. Now I filled out an incident report, but I didn’t go running and talking to the patients family about giving him two Lovenox doses’ ^39^; ‘But especially- if the patient, if there’s nothing that’s happened, for instance it happens all the time. His sodium goes down to one-nineteen okay? And nothing really happens okay? You’re not going to go in there and tell the patient that your sodium went down to one-nineteen because we didn’t do our whatever. Now if the patient asks…then you tell the patient exactly what they want to know’ ^39^; I don’t know what they had told him down in the lab and it was kind of awkward and uncomfortable when he returned because I didn’t know if i should be apologising that that had happened, or if I should just be pretending like ‘well, the doctors decided not to do that procedure. I just wasn’t going to lie to him, but I didn’t know what he had been told and there was no communication between us [the healthcare team] of what he knew’ ^16^; ‘ I don’t know that we disclose all errors. We tend to focus on that have an impact on their care’ ^43^; ‘nothing happened, so there’s no reason to bring up an issue that hasn’t been brought up and the patient won’t bring up’ ^43^; ‘There are near misses in the skies all the time. They do not get on the intercom and say you know what? We just came within 200 feet [of another plane]. But if a lot of people in the airplane see it, then they do come on and they explain it’ ^43^; ‘ I think if we were held to disclose all of those [near misses], I think that happens so often we wouldn’t have the opportunity to practise medicine’ ^35^; ‘My job is to relieve anxiety, not to create it. And to a certain extent when an error occurs that doesn’t get to the patient, it’s not their problem, it’s my problem’ ^35^; ‘You form a therapeutic alliance by being in constant communication with the patient. So to me, a medical error with no adverse event is an opportunity to form a tighter bond with the patient….if no adverse event whatsoever occurs with a medical error, I’m just delighted to tell the patient exactly what happened’ ^35^; ‘If a patient is 95 and bed-ridden, you might not want to tell them’ ^33^; ‘ It could be upsetting, they will not understand this could happen to anyone with this case.’ ^33^ | When should open disclosure take place ^37^; Attitudes and experiences concerning disclosing errors to patients ^38^; Non-disclosure ^39^; Connect the dots ^39^; It’s like walking on eggshells ^16^; Error factors ^43^; Whether to disclose near misses ^35^; Trust ^33^ |

Table 3: Reciprocal translation of findings on healthcare professionals views of the barriers and facilitators to disclosure

| Descriptor  (Groups of similar concepts clustered together/ broad thematic headings) | First order data | Second order themes |
| --- | --- | --- |
| Organisational culture | ‘The common working culture can be beneficial or also hindering. For example, if you have to fear reprisal once you disclose an error, that this falls back on a person who is then ostracised or even loses their job’ ^38^; ‘Sometimes there’s a culture of well I admit I am wrong…my employer would sack me because I’ve been open and honest and if I don’t say anything they can’t sack me’ ^41^; ‘I think there’s an openness about- we’ve caught that near miss. Give everybody a pat on the back whereas if something then bad happens, I think there’s less of an openness and then you get more into looking at well-rather than what the system did, you look at the people in the system’ ^42^; ‘As an R1 you’re starting and not many people know you and you feel as though you have to prove yourself. When you start you have a sense the nurses don’t really trust you. The parents don’t really trust you and you really feel that you have to prove that you’re competent and things like that would just turn somebody I guess, from openly talking about little mistakes and things like that because you’re afraid of, you know, perhaps the way the team will think of you, the nurses will think of you- whether they’ll talk about you’ ^42^; ‘…and I guess you’re also worried about how others perceive you or whether or not they would trust you’ ^42^; ‘I think fear kind of captures a lot of different emotions that would prevent somebody [from disclosing]…fear of being mistrusted or fear of retribution, fear of damaging career opportunities’ ^43^; ‘…one is fear of what your colleagues are going to think’ ^43^; ‘There needs to be a culture where individuals do not feel penalised for reporting errors. You should feel comfortable reporting to the chief of service of the head of nursing’ ^43^ | Barriers to disclosure ^38^; Understanding the repercussions ^41^; Degree of harm ^42^; Experience level ^42^; Reputation risk ^42^; Provider factors ^43^; Institutional factor ^43^ |
| Litigation | ‘As a matter of character. How does one approach this incident and come to terms with it. I think this is the first decisive point: will one disclose it at all or not…The person concerned will always think of themselves first’ ^38^; ‘Part of me was telling me you shouldn’t do this, why ask for trouble, this is going to just lead to litigation or complaints…but you know every time I’ve done this has been a positive and rewarding experience and I’ve not regretted it’ ^41^; ‘Through the course of my career, so many times I’ve seen very bad things have happened and patients have in the end not taken any legal action and not taken any grievance with doctors when they’ve immediately said: ‘Look I’m very sorry, this went wrong and this is why it went wrong and this is what we’re going to do to try and fix it’ ^41^; ‘I’ve learned that it’s also quite a self-preserving thing to do…the worst thing…is if they [patients] get it into their heads that there’s some sort of cover up going on, then they get the bit between their teeth and solicitors get involved and it’s all very difficult’ ^41^; ‘If you are very honest and straightforward and treat the patients right then often they feel that, they take a very generous view towards the mistake as opposed to getting very litigatous about it, which I think they are more inclined to do if there’s a big cover up and people aren’t honest’ ^41^; ‘I think there’s still a fear of the action that might be taken against you, but I think people are much more aware of, and responsible really about the failure to disclose a mistake that they’ve made…[but] there’s still a concern I guess for everyone that there will be a whole weight of something coming on them’ ^41^; ‘If families for whatever reason feel that they have not received the best medical care, they’re going to make a big stink and go to the paper and feel hard done by and I think in the situations where the families are pressing and the families raising doubts- it may be more difficult to disclose’ ^42^; ‘…two is fear of being sued and what is that going to do with your future’ ^43^; ‘You would love to be just straightforward. ‘‘Gosh, I wish I had checked that potassium yesterday. I was busy, I made a mistake, should have checked that. I can’t believe I wouldn’t do that. I will learn from my mistake and I will do better next time because this is how we learn as people’’. But if you say that to a patient, which you would like to be able to say, honestly as just another human being, is that we have this whole thing, the wait to cash in through a lawsuit’. ^35^ | Barriers to disclosure ^38^; Positive past experiences ^41^; Understanding the repercussions ^41^; Reputation risk ^42^; Provider factors ^43^; Emotional impact of error ^35^ |
| Training | ‘I have never learned (open disclosure). Can’t make facial expressions. Can’t come up with words to say… ‘I have never seen anyone do it, so I have no clue on how to do it’ ^37^; ‘…But in fact we are extremely sorry, but we just don’t know how to express or convey it’ ^37^; ‘I haven’t had any personal training. Certainly, the trust offers a sort of day if you like around breaking bad news, however I think that tends to be more related to breaking, you know, cancers and diagnoses type thing, rather than adverse events that happened ^41^; ‘My god this is really uncomfortable and I don’t have the confidence about how to do it’ ^43^; ‘I learned how to discuss grief and loss…But error? No…it’s all on-the-job’ ^43^; ‘As soon as it gets into the legal realm, suddenly as an attending physician, I feel like I need to be coached as to what can be said and how it can be said and so forth’ ^43^; We might not train our physicians enough about how to go about [disclosing error]’ ^43^; I can say right now that I do not know what the policy is’ ^43^ | Absence of disclosure education ^37^; Role models and guidance ^41^; Provider factors ^43^; Institutional culture ^43^ |
| Support | Communication is already a major focus in our training. But how do you do that when you have committed an error? This is not precise I believe. It has never been substantiated. I think that’s strange and uncomfortable for everybody’ ^38^; ‘You are told not to discuss things between yourselves and not to discuss it with the family and not to approach the family but it seems to me that actually it is better if you can speak to your colleagues ^41^; ‘If staff want to hide an error, the nuances of policy will allow them to justify it in their minds and they just won’t tell anybody’ ^16^; ‘I wouldn’t have trouble going to my charge nurse and saying that [an error] has happened because any time I’ve seen that happen [to someone else] or it’s happened to me…they’ve been very supportive’ ^16^; ‘She actually got a big lecture saying ‘you always run it by somebody before you disclose it to the families, because bedside nurses are not trained to discern litigiousness’…she felt like she did the right thing but was being told ‘don’t do that again’’ ^16^; ‘The emphasis at least in my training, has been – don’t talk about anything, keep quiet’. ^43^; The remark- Goddamn, what were you thinking? – comes out pretty often. When you get that kind of response when you admit your error, you are very unlikely to continue admitting your errors’ ^43^ | Attitudes and experiences concerning disclosing errors to patients ^38^; Clarity ^41^; Policies might help ^16^; It all depends on your nurse manager ^16^; Provider factors ^43^; Institutional culture ^43^ |
