# Supplementary figures and images for: The views and experiences of patients and health‐care professionals on the disclosure of adverse events: A systematic review and qualitative meta‐ethnographic synthesis
Source: Health Expect. 2020 Feb 19;23(3):571–83. doi: 10.1111/hex.13029 (PMC7321730; doi:10.1111/hex.13029)

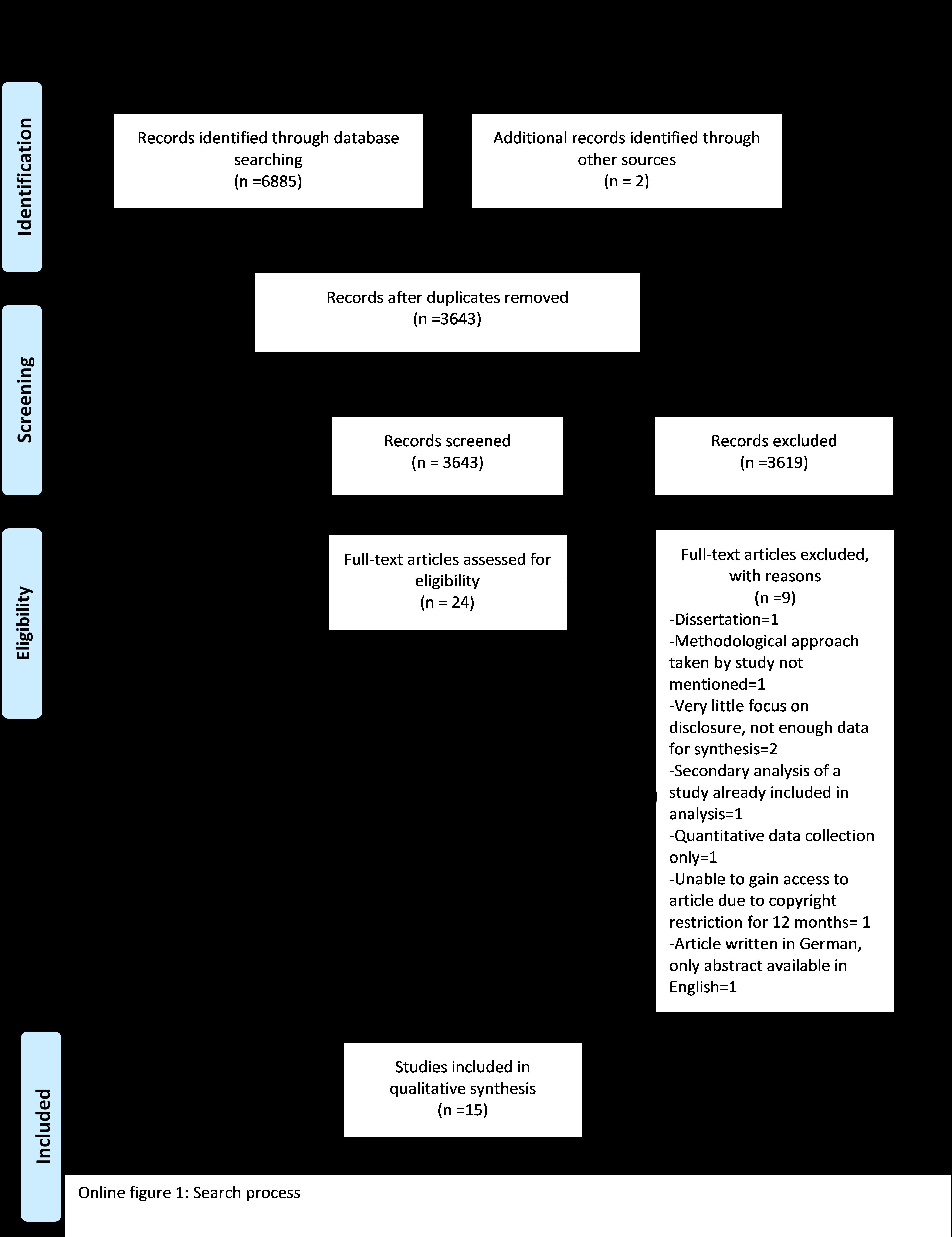

Supplement: Supplementary file 4 [file HEX-23-571-s004.jpg]
